# The study of leachate treatment by using three advanced oxidation process based wet air oxidation

**DOI:** 10.1186/1735-2746-10-1

**Published:** 2013-01-02

**Authors:** Behroz Karimi, Mohammad Hassan Ehrampoush, Asghar Ebrahimi, Mehdi Mokhtari

**Affiliations:** 1 Department of Environmental Health Engineering, School of Health, Arak University of Medical Sciences, Arak, Iran; 2 Department of Environmental Health Engineering, School of Health, ShahidSadoughi University of Medical Sciences, Yazd, Iran; 3 Environmental Research Center, School of Health, Isfahan University of Medical Sciences, Isfahan, Iran

**Keywords:** Leachate, Compost plant, Wet air oxidation, Wet peroxide oxidation

## Abstract

Wet air oxidation is regarded as appropriate options for wastewater treatment with average organic compounds. The general purpose of this research is to determine the efficiency of three wet air oxidation methods, wet oxidation with hydrogen peroxide and absorption with activated carbon in removing organic matter and nitrogenous compounds from Isfahan's urban leachate. A leachate sample with the volume of 1.5 liters entered into a steel reactor with the volume of three liters and was put under a 10-bar pressure, at temperatures of 100, 200, and 300° as well as three retention times of 30, 60, and 90 minutes. The sample was placed at 18 stages of leachate storage ponds in Isfahan Compost Plant with the volume of 20 liters, using three WPO, WAO methods and a combination of WAO/GAC for leachate pre-treatment. Thirty percent of pure oxygen and hydrogen peroxide were applied as oxidation agents. The COD removal efficiency in WAO method is 7.8-33.3%, in BOD is 14.7-50.6%, the maximum removal percentage (efficiency) for NH_4_-N is 53.3% and for NO_3_-N is 56.4-73.9%. The removal efficiency of COD and BOD_5_ is 4.6%-34 and 24%-50 respectively in WPO method. Adding GAC to the reactor, the removal efficiency of all parameters was improved. The maximum removal efficiency was increased 48% for COD, 31%-43.6 for BOD_5_ by a combinational method, and the ratio of BOD_5_/COD was also increased to 90%. In this paper, WAO and WPO process was used for Leachate pre-treatment and WAO/GAC combinational process was applied for improving the organic matter removal and leachate treatment; it was also determined that the recent process is much more efficient in removing resistant organic matter.

## Introduction

Leachate treatment is complex compound because of high concentration of COD and nitrogen [1]. Due to very high organic load and also nitro fixation inhibitory compounds, the presence of high values of free ammonium and inhibitory material like toxic compounds, biological processes are conducted with difficulty; hence, a method will be needed for leachate pre-treatment before a biological unit [2]. On the other hand, the existence of free ammonium in biological treatment is considered as a toxic agent for microorganisms; therefore, the reduction of ammonium concentration will be needed. Different methods are used for removing free ammonium [3]. For example, ammonium removal with appropriate aeration at high pH, chemical precipitation, etc. [4].

AOP and WAO processes are considered as appropriate options for wastewater treatment with average organic compounds [5]. A complete removal of organic matter is not economically possible by these methods in most cases; since final oxidation products includes acids with low molecular weight, a combination of chemical oxidation processes can be desirable with these processes [6,7]. In addition, combined processes of chemical oxidation and biological treatment will be usable for producing clean effluents [8,9]. The wet air oxidation process (WAO) is used for the reduction of severe pollution of aquatic environments to toxic material and organic matter like industrial wastewaters and sludge pre-treatment from refineries [10]. In this method, by setting an appropriate temperature and pressure and injecting determined values of oxidant (air, oxygen, hydrogen peroxide, ozone, etc.), the oxidation operation of organic matter is conducted [11]. The organic matter in a liquid phase is placed under the temperature of 100-350°C and the pressure of 5–200 bars [12]. Hydrogen peroxide and powdered or granular activated carbon can be applied for increasing the efficiency of WAO process [13].

In this study, due to high organic load and leachate ammonium of the Compost Plant, the wet air oxidation method and WAO catalytic methods like wet oxidation method with (WAO) hydrogen peroxide and WAO/GAC combined process were applied. The advantages of these methods are to breaking organic material and increase a biodegradation capability and to reduce the effluent toxicity [14]. The purpose of this research is to determine the efficiency of three methods, including wet air oxidation, wet oxidation with hydrogen peroxide, and the absorption process in removing the organic matter as well as nitrogenous compounds from urban Leachate of Isfahan composting factory.

## Materials and methods

**Figure 1:**
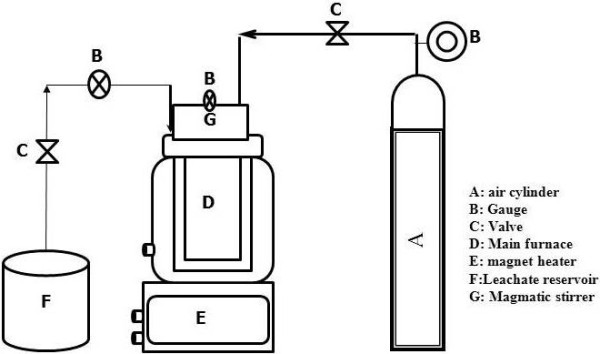
Wet peroxide oxidation reactor system.

WAO method: a reactor with the volume of three liters was used which is able to bear a pressure to 100 bars and equipped with a pressure drain valve, monometer, an injection pipe, a sample output, etc. (manufactured by Combine Company of Arak). Leachate sampling is taken from an evaporative lagoon. The prepared sample with the volume of 1.5 liters was entered into a steel (stainless) reactor. Study was conducted at three temperatures of 100, 200, and 300°C as well as three retention times of 30, 60, and 90 min. To determine the pressure, an initial pilot study was done; then a 10-bar pressure was selected as the best one. A schematic of the WAO process is in Figure 1.

WPO method: the used reactor for WPO method also includes similar characteristics. An oxidation reaction with hydrogen peroxide was performed in the reactor under a high pressure (10 bars). The only difference of this method with WAO is in creating acidic conditions of the environment before the sample entry into the reactor. After injecting 1.5 liters of the sample into the reactor per liter of the sample, the volumes of 1, 2.5, and 5 mL of hydrogen peroxide (30% w/v) was added to the reactor as an oxidant. After passing the test steps, and the reactor cooling, parameters were measured. How to operate the reactor with WPO method is available in Table 1.

**Table 1:** Wet Peroxide oxidation operational conditions

**Oxidizing agent**	**pure oxygen and H**_**2**_**O**_**2**_
Partial Pressure of O_2_	10 bar
Temperature	100, 200 and 300°C
Volume of reactor and sample	3 L and sample Volume 1500 CC
Duration of the reactions	1.5 hours after preheating period
Reaction time	30, 60 and 90 min
Cooling of reactor	2–3 h
Mixing inside reactor	50 S^-1^

WAO/GAC method: a reactor used in this method has similar characteristics with WAO, and 2 g/L value of the granular activated carbon was added to the reactor. For getting further results, pH solution on 5.5 was set in accordance to the previous study [15]. Prior to GAC entry into the reactor, it was heated at the temperature of 105°C for 24 hours in the oven, and then was entered into a desiccator. After doing reactions in the reactor, a centrifuge with 6000 rpm per 15 minutes was applied to separate GAC prior to chemical analyses. All the steps were taken to prepare the granular activated carbon in accordance to the study of Klç et al. [15].

Chemical: The iron sulfate (FeSO_4_.7H_2_O), H_2_O_2_ (30% W/V), H_2_SO_4_, NaOH, acetic acid (CH_3_COOH), potassium dichromate (K_2_Cr_2_O_7_), HgSO_4_, Ag_2_SO_4_, manganese oxide and powder and granular activated carbon were purchased from Merck, Germany.

Studied parameters: in order to study the reactor performance and compare three oxidation processes, (BOD5 and COD) organic load values, ammonium and nitrate were performed in terms of Standard Methods 5220-C and 5210-B [16]. COD measurement was conducted based on Dichromate method (closed reflux, 5220C, colorimetric method), and BOD_5_ in accordance to Winkler's method (5210 B) (APHA, 2005). In addition, to measure ammonia and nitrate concentrations, a spectrophotometer DR/2000 was used in accordance to EPA method (960 and 351). Other parameters like pH, temperature, and EC were also measured before and after the reaction using a pH device (with a model of 520-HACH) based on APHA Standard Methods.

All data were subjected to two-way analysis of variance (ANOVA), paired and independent sample T-test and Pearson correlation using SPSS Version 14. Statistical significance was tested using Confidence Interval 95%. The results are shown as mean with excel Version 2010.

## Results

The study was conducted at three temperatures 100, 200, and 300°C and the retention time 30–90 min. In Tables 2 and 3, the removal efficiency of different parameters has been taken in to consideration by all three parameters in different temperatures and retention times.

**Table 2:** **Effects of changes in temperature, retention time and H**_**2**_**O**_**2**_**volume on the COD and BOD removal efficiency (%) in WPO process**

**Parameter**	**H**_**2**_**O**_**2**_**volumes**	**1 mL**			**2.5 mL**			**5 mL**		
	**Temperature time**	**100°C**	**200°C**	**300°C**	**100°C**	**200°C**	**300°C**	**100°C**	**200°C**	**300°C**
COD	30 min	4.66	4.5	16	15	21.7	19.5	20.6	10.8	21.3
	60 min	6.6	27.5	24.8	23.6	32.8	26	29	33.7	30
	90 min	12	42	34	31.5	44	36.3	32	34	39.4
BOD	30 min	39	32.5	20.7	25.7	10.4	39	24	31.5	32
	60 min	35.7	26.3	39.7	29.9	21.5	40.5	37	35	36.8
	90 min	30	34	45	36.5	32	44	40	42	50
NH_3_	30 min	68.1	54.5	54	53.5	56	56	58	35	34
	60 min	57.5	57	69	48.5	64	61	65.5	28	28
	90 min	29	58	41	49	65	56	79.5	25	25

**Table 3:** Effects of changes in temperature and retention time on the COD and BOD removal efficiency (%) in WAO and WAO/GAC methods

**Parameter**	**Methods**	**WAO**			**WAO/GAC**		
	**Temperature time**	**100°C**	**200°C**	**300°C**	**100°C**	**200°C**	**300°C**
COD	30 min	7.8	12.6	19	35	41	43.5
	60 min	15.2	16.2	24.6	33	41.7	46
	90 min	24	28	33	41.5	48	56
BOD	30 min	44.5	47	50.6	38	35.5	43.5
	60 min	31	41.3	46	35	35	41.5
	90 min	15	25.5	38.5	31	33	35
NH_3_	30 min	6	29.4	31.5	37	58.4	63
	60 min	9.6	42.43	50.9	42.5	52	58
	90 min	15.4	47.61	54.6	53	64.6	68

**WAO process and WAO/GAC combined processes:** in Tables 2 and 3, the effect of changes in temperature and retention time on the removal efficiency of two COD and BOD parameters is available in three methods WAO, WAO/GAC and WPO. Table 3 indicates the removal efficiency (%) of different parameters by the wet oxidation process with pure oxygen as well as WAO/GAC combined process in different temperatures and retention times.

**WPO process:** the maximum removal efficiency of COD (39.5) was obtained in the temperature of 300°C, retention time of 90 minutes and volume of H_2_O_2_, 5 mL (Table 2). In Table 2, the removal efficiency (%) of different parameters is available by the wet oxidation process with hydrogen peroxide (WPO) in different temperatures and retention times. In the temperature 100°C, the removal efficiency of ammonium is 68% at the volume of 1 ml peroxide and retention time of 90 minutes which is the most efficiency. The most production efficiency of this parameter is 14.6% in the temperature of 300°C, retention time of 90 minutes, and peroxide volume of 5 ml. Also efficiency has been increased in 5 ml peroxide and 60 minutes so that the average efficiency is 19.7%.

**Figure 2:**
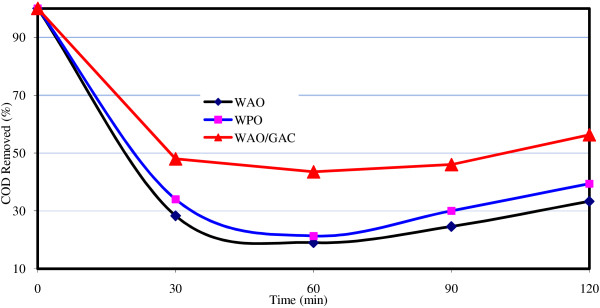
The comparison of the removal efficiency of three methods WAO, WPO and WAO/GAC in COD removal.

**Figure 3:**
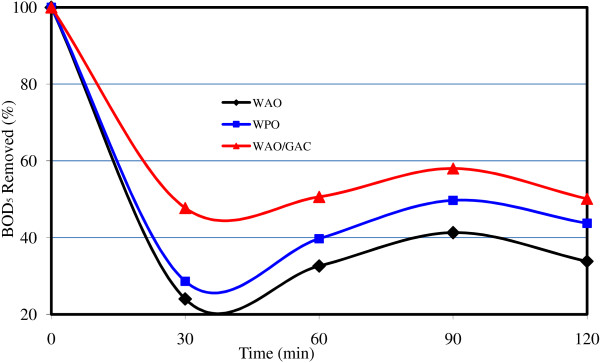
The comparison of the removal efficiency of three methods WAO, WPO and WAO/GAC in BOD removal.

**Figure 4:**
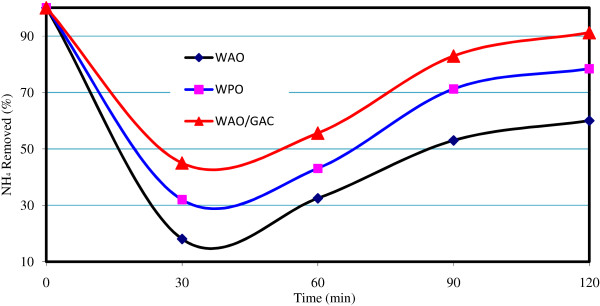
**The comparison of the removal efficiency of three methods WAO, WPO and WAO/GAC in NH**_**3**_**removal.**

In Figures 2, 3, 4, the comparison of the removal efficiency of three methods WAO, WPO and WAO/GAC is available in COD, BOD and NH_3_-N removal.

## Discussion

**WAO process:** because of refractory organic compounds in the leachate, biodegradation capability is usually low. To improve the degradation capability, WAO process was applied to pre-treatment [17]. According to the presence of refractory compounds in the leachate, COD removal efficiency was obtained low. Average COD removal efficiency in WAO process is 7.8-33.3%. This method is unable to remove the entire organic load in a limited time. Due to the degradation of heavy compounds and large molecules of organic compounds to simpler ones, the COD value is increased; and consequently despite desired efficiency of the process, the efficiency of the reactor appears low [18]. During the process, it may create intermediate compounds, including volatile fatty acids (VFAs). Perhaps, the reason for pH reduction after the process is the same matter so that it was reduced from the average value of 7–8 in the input to 5–6 [19]. The average value of NH_4_-N was changed from 180.2 to 84–300 g/L in the raw leachate. NO_3_-N was also reduced from 578 mg/L to the values of 150.6-252 mg/L. The maximum removal percentage (efficiency) of NH_4_-N was obtained 53.3% and 56.4-73.9% for NO_3_-N. In general, the average removal percentage of NO_3_-N is 65% in this process. Huang et al. (2001) observed that with the increase of temperature to more than 300°C, the ammonium production is increased and pH decreased [3].

**WPO process:** this method has been used in many industrial processes, such as food industry wastewater [20], color removal from textile industry, sludge wastewater treatment, pharmaceutical industry treatment, production of chemicals, organic matter degradation, cellulosic compounds of water steam pre-treatment produced from processes of food processing, sugar refinement, coffee production, etc. [21]. Due to the use of hydrogen peroxide at a high temperature and pressure, oxidation power and the formation of oxidizing radicals are increased. Using this process will result in the improvement of COD removal efficiency. The removal efficiency of the process in COD removal was increased to 4.6-11.95% at 100°C, and to 16-34% at 300°C at the volume of 1 ml hydrogen peroxide. The COD removal efficiency was increased to 4.6-11.95% at 100°C and the maximum used volume of hydrogen peroxide was 20–32.2% and increased to 21-39% at 300°C. The removal efficiency of BOD_5_ also reached 23.9-40% at 100°C and 31.9-50% at 300°C at the volume of 5 mL hydrogen peroxides. The BOD_5_ removal efficiency was increased compared to the previous stage (Table 2), but it is observed, the efficiency is more at 300°C. In a study by Rodruez, E.M et al. for atrazine degradation that the wet oxidation method with hydrogen peroxide was applied, it was seen that with increasing the volume of injected hydrogen peroxide, the organic matter degradation was added. Oxygen injection in these conditions was also added to the work efficiency. Results of this study confirm the recent consideration too [22].

The results of a study by Jing et al. [23] showed that WPO can reduce the organic compounds of oil sludge effectively; in addition, the retention and reaction temperature were regarded as the important factors for removing COD. At the reaction temperature of 340°C, the initial volume of oil field sludge 4000 mg/L, retention time of 9 minutes, and the COD removal efficiency reached 88.6%. The COD removal efficiency was increased by the increase in the reaction temperature and retention time [23].

**WAO in combination with GAC:** adding GAC to the reactor, COD removal efficiency was improved. The maximum removal efficiency of COD was 48% by this method; whereas, the maximum removal efficiency of WAO is only 33%. As it is observed from Figures 2, 3, 4, the most COD removal efficiency is concerned with WAO/GAC combined process.

The disadvantages of the method will be high consumption of energy and GAC that need was regenerated; however, among the three studied methods, this one is more appropriate for removing the organic matter and ammonium. The BOD_5_ removal efficiency is 31–43.6%, and the ratio of BOD_5_/COD was also increased to 90%. Some researchers know WAO combined method with GAC activated carbon as an appropriate option for the leachate treatment due to synergist property [24].

WAO enables the initial oxidation of organic compounds to more stable oxidized state, also decomposition of large and resistant pollutants to small molecules, and as a result, CO_2_ and H_2_O formation of water; whereas, GAC results in increasing the reaction rate in a degradation process through the formation of H_2_O radicals. It reacts quickly with target compounds in the leachate via OH radicals and gives rise to final degradation of the leachate. Increasing the oxidation of organic compounds by WAO in the leachate is dependent on the initial value of GAC and reactions chain of WAO degradation. In this case, WAO by affecting the decomposition of molecules will result in the absorption increase in pyrrolic groups in a graphenic layer in GAC (the same basal level of electrons), and due to the reduction in microporous congestion, the absorption power will be increased. In addition, to regenerate the activated carbon, WAO method can be applied [25].

The regeneration of activated carbon is performed by heating carbon at the temperature of 700° to 900°C. As a result, energy costs and large amounts of carbon are lost due to heat. The regeneration of activated carbon by WAO is more appropriate than thermal regeneration of the activated carbon. Results of this operation in a laboratory scale indicated that due to increased breakdown of the organic compounds to simpler ones at higher temperatures, the use of this method along with a biological treatment (aerobic and anaerobic) can be a promising option for the leachate treatment of the compost plant [26].

Also, given that no similar work has been done using the wet oxidation method, therefore, it is required that other researchers apply the results of this plan or method in a semi-industrial and full scale. The problems of this study include an accurate setting of pressure and temperature in the reactor, the possibility of bursting pipes and fittings during work, etc.

## Conclusion

The study considers the wet air oxidation process to reduce organic load from the composting factory leachate. The WAO process is very effective in oxidizing high concentrations of organic matter to obtain more than 35% and 38% removal efficiency of COD and BOD_5_. Using WPO process will result in the improvement of COD removal efficiency. The removal efficiency of the process in COD removal was increased to 4.6-11.95% at 100°C, and to 16-34% at 300°C at the volume of 1 ml hydrogen peroxide. The removal efficiency of BOD_5_ also reached 23.9-40% at 100°C and 31.9-50% at 300°C at the volume of 5 mL hydrogen peroxides. Adding GAC to the reactor, COD removal efficiency was improved. The maximum removal efficiency of COD was 48% by this method; whereas, the maximum removal efficiency of WAO is only 33%. Results of this operation in a laboratory scale indicated that due to increased breakdown of the organic compounds to simpler ones at higher temperatures, the use of this method along with a biological treatment (aerobic and anaerobic) can be a promising option for the leachate treatment of the compost plant.

## Competing interests

The authors declare that they have no competing interests.

## Authors’ contributions

This work is part of the Master thesis of BK that MHE developed initial idea and proposed and supervised the whole work. BK administered data collection. AE and MM are Faculty advisor of thesis. All authors read and approved the final manuscript.
